# First report of cavitary pneumonia due to community-acquired *Acinetobacter pittii*, study of virulence and overview of pathogenesis and treatment

**DOI:** 10.1186/s12879-017-2589-0

**Published:** 2017-07-06

**Authors:** Romaric Larcher, Alix Pantel, Erik Arnaud, Albert Sotto, Jean-Philippe Lavigne

**Affiliations:** 1 Department of Internal Medicine, Caremeau University Hospital, 29 place du Professeur Debre, Nimes, France; 2 Department of Microbiology, Caremeau University Hospital, 29 place du Professeur Debre, Nimes, France; 3 Department of Infectious Diseases, Caremeau University Hospital, 29 place du Professeur Debre, Nimes, France

**Keywords:** Community-acquired pneumonia, Cavitary pneumonia, Cavitation, *Acinetobacter pittii*, *Acinetobacter calcoaceticus-baumannii* complex, Virulence, Biofilm

## Abstract

**Background:**

*Acinetobacter pittii* is a nosocomial pathogen rarely involved in community-acquired infections. We report for the first time that *A. pittii* can be responsible for cavitary community-acquired pneumonia and study its virulence, and discuss its pathogenesis and treatment options.

**Case presentation:**

A 45-year-old woman with a history of smoking and systemic lupus was admitted to Nimes University Hospital (France) with coughing and sputum lasting for three weeks. Thoracic CT scanner showed cavitary pneumonia. Broncho-alveolar lavage cultures found community-acquired *Acinetobacter calcoaceticus-baumannii* complex. The clinical outcome was favourable after twenty-one days of antimicrobial treatment by piperacillin/tazobactam and amikacin then cefepime. Multilocus sequence typing (MLST) analyses identified an *A. pittii* ST249. Despite the atypical clinical presentation with an unexpected partial destruction of lung parenchyma, we found very low virulence potential of the *A. pittii* strain with nematode killing assays and biofilm formation test. The median time required to kill 50% of the nematodes was 7 ± 0.3 days for *A. pittii* ST249, 7 ± 0.2 days for *A. baumanii* NAB ST2 and 8 ± 0.2 days for *E. coli* OP50, (*p > 0,05*). *A. pittii* ST249 showed significantly slower biofilm formation than *A. baumanii* NAB ST2: BFI = 8.83 ± 0.59 vs 3.93 ± 0.27 at 2 h (*p < 0.0001*), BFI = 6.3 ± 0.17 vs 1.87 ± 0.12 at 3 h (*p < 0.0001*) and BFI = 3.67 ± 0.41 vs 1.7 ± 0.06 after 4 h of incubation (*p < 0.01*).

**Conclusions:**

Community-acquired *A. pittii* should be considered as possible cause of sub-acute cavitary pneumonia particularly in a smoking and/or immunocompromised patient despite its low virulence potential.

## Background

The classic underlying causes of lung abscess or cavitary lung lesions are anaerobes, *Streptococcus pneumonia, Streptococcus milleri* group, *Staphylococcus aureus* and *Klebsiella pneumoniae*. More rarely *Pseudomonas aeruginosa* and other aerobic Gram-negative bacilli, *Nocardia* spp., *Aspergillus* spp. and *Cryptococcus* spp. could be identified [1]. To our knowledge, *Acinetobacter pittii*, formerly known as *Acinetobacter* genomospecies 3, has not been described as a cause of cavitary pneumonia before and it appears to be an unusual cause of community-acquired infections [1–3]. However, it is increasingly described as a cause of hospital-acquired infection particularly in intensive care unit setting [4]. Here, we report the first case of cavitary community-acquired pneumonia due to *A. pittii* in a patient with a systemic lupus and study the virulence profile of *A. pittii*.

## Case presentation

A 45-year-old woman, was admitted to Nimes University Hospital (France) for a cough and sputum lasting for three weeks with an unfavourable outcome despite prescription of antibiotics (cefixime then pristinamycin). She had a medical history of smoking and systemic lupus diagnosed in 1991 with moderately reduced glomerular filtration rate (57 ml/min/1.73m^2^), stroke and right nephrectomy in 2011. Her usual treatment included hydroxychloroquine, clopidogrel and atorvastatine.

**Fig. 1:**
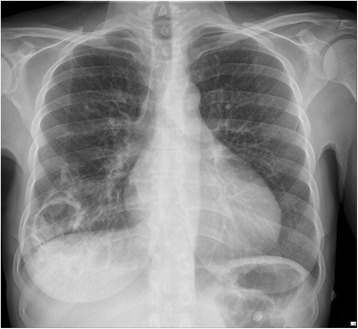
Chest X-ray showing a community-acquired pneumonia due to *A. pittii* with a large pulmonary cavity of the right lower lobe, at hospital admission

**Fig. 2:**
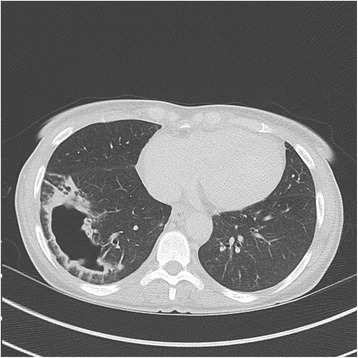
Thoracic CT scan showing a cavitary community-acquired pneumonia due to *A. pittii* with the largest cavitation of the right lower lobe, three days after admission

**Fig. 3:**
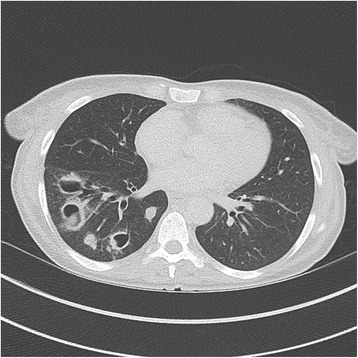
Thoracic CT scan showing a cavitary community-acquired pneumonia due to *A. pittii* with the three others cavitations and nodules of the right lower lobe, three days after admission

At admission, she presented dyspnoea, crackles of the right lung field and normal temperature. The white blood cell count was 19 × 10^3^ cells/μL and C reactive protein was high at 144 mg/L. Chest X-ray showed a large pulmonary cavity of the right lower lobe (Fig. 1) and computed tomography (CT-scan) showed four large cavities and nodules of the right lower lobe (Figs. 2 and 3). Blood cultures remained negative. A fibroscopy with broncho-alveolar lavage (BAL) showed local inflammation with sputum. Piperacillin/tazobactam plus amikacin was administered as empirical treatment. The BAL culture was positive with more than 10^4^ CFU/mL of Gram negative bacilli.

MALDI-TOF (Matrix Assisted Laser Desorption Ionisation/Time Of Flight) analysis (Vitek MS, bioMérieux) identified *A. calcoaceticus-baumannii* complex (99%). Antimicrobial susceptibility was tested by disc diffusion in accordance with EUCAST (European Committee on Antimicrobial Susceptibility Testing) recommendations (version 5.0, 2015; http://www.eucast.org). The isolate was susceptible to ticarcillin, piperacillin, third and fourth generation cephalosporins, imipenem, ciprofloxacin and amikacin. Cultures for mycobacteria, *Nocardia* spp., *Aspergillus* spp*.* and *Cryptococcus* spp. were negative. Bronchial biopsy was normal.

Cavitary community-acquired pneumonia due to *A. calcoaceticus-baumannii* complex was considered. The antimicrobial treatment was changed to cefepime after seven days of piperacillin/tazobactam and two days amikacin. After twelve days of antimicrobial treatment (including five days cefepime) the CT-scan showed persistent excavated lung lesions. Size of the largest cavity was slightly reduced and the walls were thinner. The clinical outcome was favourable after twenty-one days of antimicrobial treatment (including fourteen days cefepime). The Positon Emission Tomography - Computed Tomography performed at the end of the antimicrobial therapy did not find hypermetabolic fixation. Six months later, CT-scan showed residual excavated lung lesions of the right lower lobe. The bronchoscopy with biopsies was normal and the BAL cultures were negative.

### Species identification

As species included in *A. calcoaceticus-baumannii* complex are closely related, rpoB sequencing and multilocus sequence typing (MLST) (bigsdb.web.pasteur.fr) [5] were performed to characterize the taxonomic status of the strain and the clone involved in this case. The isolate was identified as *A. pittii* belonging to ST249.

### Virulence study

To evaluate the virulence of the *A. pittii* strain, we conducted a *Caenorhabditis elegans* nematode killing assay, a virulence model well described to study *Acinetobacter* spp. virulence [6]. The median time required to kill 50% of the Fer-15 *C. elegans* population (LT50) was compared between *A. pittii* isolate and an *A. baumannii* strain belonging to international clone II/ST2 isolated in our laboratory (NAB ST2). In vivo kinetics of killing of *C. elegans* infected by *A. pittii* and NAB ST2 were compared with the survival curve for worms fed on non-pathogenic *E. coli* (OP50) using a log-rank (Mantel-cox) test.

**Fig. 4:**
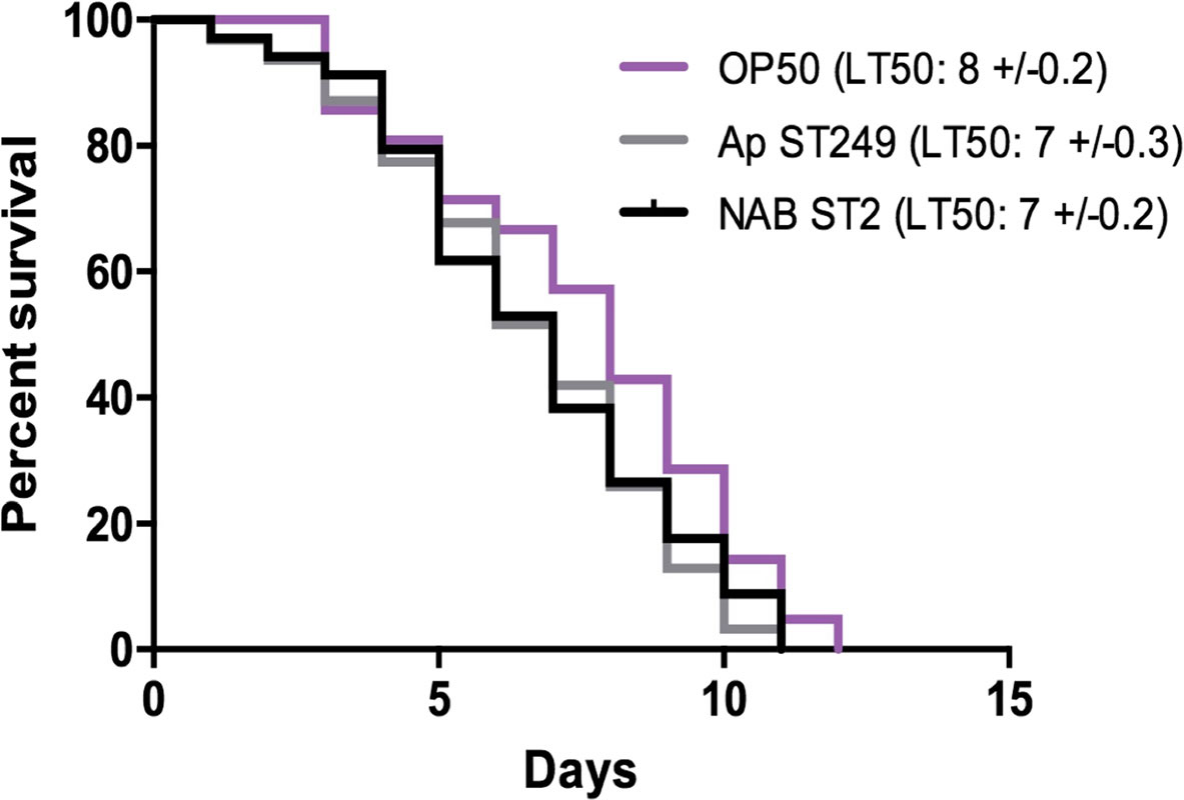
In vivo kinetics of killing of *C. elegans* infected by *A. pittii* ST249 (Ap ST249) and *A. baumannii* ST2 (NAB ST2) compared with the survival curve for worms fed on non-pathogenic *E. coli* (OP50) using a log-rank (Mantel-cox) test to evaluate differences in survival rates between the different strains (*p* non-significant)

There was no significant difference between kinetics of killing of *C. elegans* infected by *A. pittii* ST249 and NAB ST2 compared with those fed on non-pathogenic *E. coli* OP50, showing a very low virulence potential for the two strains equivalent to the standard feeding strain *E. coli* OP50 for nematodes (Fig. 4). The LT50 were 7 ± 0.3 days, 7 ± 0.2 days and 8 ± 0.2 days respectively for *A. pittii* ST249, NAB ST2 and *E. coli* OP50, (*p > 0,05*).

Given that biofilm formation is reported to favour bacterial colonization and persistence, biofilm-forming ability was investigated and kinetics of *A. pittii* ST249 and NAB ST2 were compared. The kinetics of the early phase of biofilm formation for *A. pittii* and NAB ST2 were studied using the BioFilm Ring Test® (BioFilm Control, Saint Beauzire, France) [7]. A high BioFilm Index (BFI) value (>8) corresponds to high mobility of free beads under magnetic attraction (no biofilm), while a low value (≤2) corresponds to complete immobilisation of beads (strong biofilm) [8]. BFI values between 2 and 8 indicate that biofilm formation is in progress. Three experiments with two repeats were performed per strain and per incubation time. Mean BFI were compared between each strain after 1, 2, 3, 4 and 5 h, using two-way Anova test followed by Bonferroni’s multiple comparisons test. Statistics and graphs were prepared using the software package GraphPad Prism 6.0®.

**Fig. 5.**
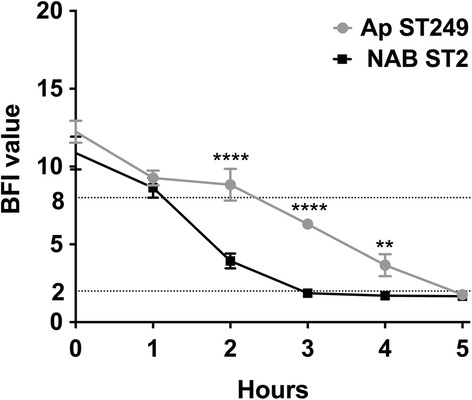
Comparison between the kinetics of the early phase of biofilm formation for *A. pittii* ST249 (Ap ST249) and *A. baumannii* ST2 (NAB ST2). Means ± standard deviations of BioFilm Indice (BFI) for at least three independent replicates are presented and significant differences between each strain at each time using two-way Anova test, followed by Bonferroni’s multiple comparisons test are indicated by ** (*p < 0.01*) and **** (*p < 0.0001*)

There were no significant differences between the two strains at the very beginning of kinetics (BFI = 9.27 ± 0.27 for *A. pittii* and 8.63 ± 0.37 for NAB ST2 at 1 h of incubation, *p > 0,05*) (Fig. 5). However, at the next time points, *A. pittii* ST249 showed a significantly slower biofilm formation than NAB ST2: BFI = 8.83 ± 0.59 vs 3.93 ± 0.27 respectively at 2 h (*p < 0.0001*), BFI = 6.3 ± 0.17 vs 1.87 ± 0.12 at 3 h (*p < 0.0001*) and BFI = 3.67 ± 0.41 vs 1.7 ± 0.06 after 4 h incubation (*p < 0.01*). *A. pittii* biofilm was constituted after 5 h of incubation (BFI = 1.77 ± 0.09).

## Discussion and conclusions

We report the first case of cavitary community-acquired pneumonia due to an *A. pittii* strain with low virulence potential, in a smoking patient with systemic lupus.

*A. pittii* is a member of the *A. calcoaceticus-baumannii* complex which also includes *A. baumannii*, *A. calcoaceticus* and *A. nosocomialis*. As a result of advances in molecular biology, *A. pittii* is increasingly recognized as a significant cause of nosocomial infection, particularly in intensive care unit setting, but nevertheless remains uncommon [3]. This bacterium is rarely observed to cause community-acquired infections notably cavitary pulmonary disease [1]. *A. pittii* ST249 has been described only twice, in two patients with hospital-acquired infection in Germany, a country where the prevalence of *A. pittii* seems high among species of *A. calcoaceticus-baumannii* complex [9]. The proportion of *A. pittii* community-acquired infections is not defined because of technological limitations in species identification across laboratories worldwide [5, 10]. Prevalence may also be under-evaluated in healthy individuals because the low virulence profile does not result in clinical disease.

The large destruction of lung parenchyma with both cavitary lung lesions and pulmonary nodules in this case suggests that *A. pittii* is highly virulent. However, the *C. elegans* killing assays and the BioFilm Ring Test® showed very low virulence potential and a poor ability to form biofilm as recently observed [11, 12]. The significant variability of virulence described among *A. baumannii* complex species probably explains the sub-acute clinical course unlike the fulminant evolution usually associated with *Acinetobacter* community-acquired pneumonia in tropical areas [12–14]. Moreover, the mortality rate for patients infected with *A. pittii* seems to be lower than for *A. baumannii* (15% versus 40%) [4, 10]. Finally, the hosts’ immune status and tobacco consumption seem to play a crucial role to facilitate infection. *A. pittii* and *A. baumannii* appear genetically and metabolically similar [15] and probably share the same risk factors for causing community-acquired infections: smoking, excessive alcohol consumption, diabetes mellitus and chronic lung disease [13, 14]. In this case, the patient is diabetic, smokes and also has lupus, a condition known to increase the risk of infection with or without immunosuppressive drugs [16]. *A. pittii* is widely distributed in the environment and may contaminate food and animals, thus humans could acquire skin and/or oral carriage which subsequently favours infection [13, 17]. A study with more patients would allow us to better characterize the virulence of *A. pittii* and the host-pathogenic interaction but seems difficult due to the low prevalence of this type of infection.

With regard to drug choice, on the one hand, initial empiric antimicrobial therapy may be inappropriate if following standard practice guidelines for community acquired pneumonia, except if a fluoroquinolone is chosen [18]. On the other hand, second line treatment is ultimately most often appropriate because *A. pittii* is relatively susceptible to antibiotics used in this case [4, 6]. The most effective drugs are carbapenems [5, 10]. Piperacillin/tazobactam, ticarcillin/clavulanate, ceftazidime or fluoroquinolone are alternative choices [10, 19]. However, carbapenem resistance does occur frequently in the *A. calcoaceticus-baumannii* complex [5] and *A. pittii* ST249 has been described in association with the production of a carbapenemase GIM-1 [9]. In these cases, amikacin or colistin often remain the only therapeutic option.

In conclusion, one case of *A. pittii* community-acquired pneumonia revealed by cavitary lung lesions with a sub-acute clinical course lead to describing its low virulence profile. In the setting of immunocompromised patients and tobacco consumption, evaluation of *A. pittii* as a possible cause of community-acquired pneumonia should be considered, particularly in case of unsatisfactory response to first line antimicrobial therapy. The clinical course seems to be less severe than when caused by *A. baumannii*. As we can now more easily identify *A. pittii* in the *A. calcoaceticus-baumannii* complex, studying more patients could be interesting to asses the clinical features of this type of pneumonia.

## Abbreviations

BAL: Broncho-alveolar lavage
BFI: BioFilm Indice
CFU: Colony Forming Unit
CT-scan: Computed tomography
EUCAST: European Committee on Antimicrobial Susceptibility Testing
LT50: Median time required to kill 50% of the Fer-15 *C. elegans* population
MALDI-TOF: Matrix Assisted Laser Desorption Ionisation/Time Of Flight
MLST: MultiLocus Sequence Typing
NAB ST2: *A. baumannii* strain belonging to international clone II/ST2
OP50: Non-pathogenic *E. coli*
